# Twenty year outcomes following short-segment posterior instrumentation and fusion for thoracolumbar burst fractures: A retrospective observational study

**DOI:** 10.1097/MD.0000000000040579

**Published:** 2024-11-15

**Authors:** Yigit Kultur, İlker Sarikaya, Mahmut Kursat Ozsahin, Cumhur Deniz Davulcu, Onder Aydingoz

**Affiliations:** a Yeni Yuzyil University Gaziosmanpasa Hospital, Orthopedics and Traumatology, Istanbul, Turkey; b Ortopediatri Clinic, Orthopedics and Traumatology, Istanbul, Turkey; c Cerrahpasa Medical Faculty, Orthopedics and Traumatology, Istanbul, Turkey.

**Keywords:** spine, spine fractures, vertebra

## Abstract

This study reviews the long-term efficacy of short-segment posterior instrumentation and fusion (SSPIF) in treating thoracolumbar burst fractures. Authors retrospectively reviewed the radiographic results of single-level thoracolumbar burst fractures treated by SSPIF. Vertebral body height and wedge angles were measured on the preoperative, postoperative, and follow-up radiographic images. The degree of pain and work ability was measured using the Denis scale. The analysis consisted of 12 patients with a mean age of 39.7 years (range 21–60) and a mean follow-up of 225.6 ± 20.3 months. There were significant differences among the wedge angles at preoperative and other periods of time, but there was no significant difference between the early postoperative and all other time periods afterwards (*P* < .001, *P* = .567, *P* = .937, *P* = .879). SSPIF effectively restored the anterior and middle vertebral body height and wedge angle deformities, and the improvement was maintained for almost 20 years after the surgery. Therefore, SSPIF is a safe and effective modality of treatment for thoracolumbar burst fractures.

## 1. Introduction

Traumatic spinal column injuries most classically involve the thoracolumbar junction area, specifically the T11-L2. It is a biomechanically weaker region owing to the transition from a relatively rigid thoracic spine to a more mobile lumbar spine. Burst fractures, classically sustained from high-energy axial loading, have been variously reported to account for 21% to 58% of all thoracolumbar fractures.^[1–4]^ The modality of treatment has now firmly been established wherein surgical intervention, particularly short-segment posterior instrumentation and fusion (SSPIF) can be done in thoracolumbar burst fractures with the help of pedicle screws that are placed 1 level above and 1 level below the fracture site by fusion, thereby affording long-term stability to the segment.^[5,6]^

SSPIF offers several advantages, including enhanced initial stability, early pain-free mobilization, preservation of spinal segment mobility, reduced operative time, minimal blood loss, and a less invasive approach than longer segment fixation strategies.^[7–12]^ Nonetheless, SSPIF is associated with various complications, including long-term reduction loss, implant failure, screw loosening, kyphotic collapse, and chronic pain.^[13–16]^

However, until now, there has been a lack of evidence-based guidelines or consensus regarding the management of burst thoracolumbar spinal fractures.^[5,8,17,18]^ Except for SSPIF, other options for surgery tried in the literature are long-segment posterior instrumentation and fusion (LSPIF) and anterior decompression with fusion, along with hybrid techniques that involve a combination of both of the above approaches.^[19–22]^ All these techniques have their proponents, but very often the choice of treatment is based on fracture characteristics, the general condition of the patient, and experience of the surgeon. A comparison of the results would be interesting, particularly by identifying different techniques for refining optimum therapeutic strategies in thoracolumbar burst fractures, especially when SSPIF is inadequate to avoid long-term complications.

In view of the complexity and possibility of serious consequences of thoracolumbar burst fractures, the results of surgical techniques must be constantly reassessed. This is a retrospective observational study to analyze long-term outcomes of short-segment posterior instrumentation and fusion (SSPIF) in patients with thoracolumbar burst fractures. We hypothesized that the SSPIF technique would maintain vertebral stability and the satisfactory surgical outcomes over a nearly 20-year follow-up period. This study highlights the lack of long-term studies in the literature regarding the effectiveness of SSPIF for managing thoracolumbar burst fractures. The data collected nearly 20-year follow-up period provide valuable insights into the long-term outcomes of SSPIF.

## 2. Methods

### 2.1. Study design

We retrospectively reviewed all consecutive adult patients treated at a single university hospital for single-level thoracolumbar (T11-L5) burst fractures without neurological deficits, who were treated surgically with 4-screw constructs (SSPIF) between 1997 and 2002 within 3 weeks from the time of injury. This study was approved by the local ethics committee (Istanbul University Cerrahpasa – Cerrahpasa Medical Faculty Clinical Research Ethics Committee – Approval Number: 26052009-297). Informed consent was obtained from all the patients participating in this study.

The investigators reviewed the patients’ charts. The time elapsed until surgery and the length of hospital stay were recorded. Pain and functional status were evaluated by 1 independent observer according to the Denis pain and work scales postoperatively in 2004, 2010, and at the last visit.^[23]^ Patients with polytrauma, pathological fractures, multilevel injuries, and gross osteoporosis were excluded.

### 2.2. Surgical technique

This is a well-established posterior approach in thoracolumbar vertebrae and is widely recognized as the standard surgical technique. Steps to this procedure include appropriate positioning of the patient, hyperextension of the thoracolumbar junction, and a posterior midline approach extending 1 vertebra above and below the level of the fracture. Four screws were inserted into the vertebrae situated cranial and caudal to the fractured segment. Under C-arm guidance, long monoaxial pedicle screws are typically inserted to ensure that they are parallel to the endplates of the intact vertebrae, aiming to correct post-traumatic kyphosis at the fracture site.

Using pre-contoured rods, the fractured vertebra is pushed forward, facilitating the realignment of the sagittal plane of the spine. These rods were secured with transverse connectors at 2 levels, providing robust stabilization in the sagittal, frontal, and rotational planes. The facet joints and posterior vertebral arches were decorticated and bone grafts were applied posteriorly and posterolaterally. All procedures were carried out by the same surgeon.

### 2.3. Radiological studies

**Figure 1.**
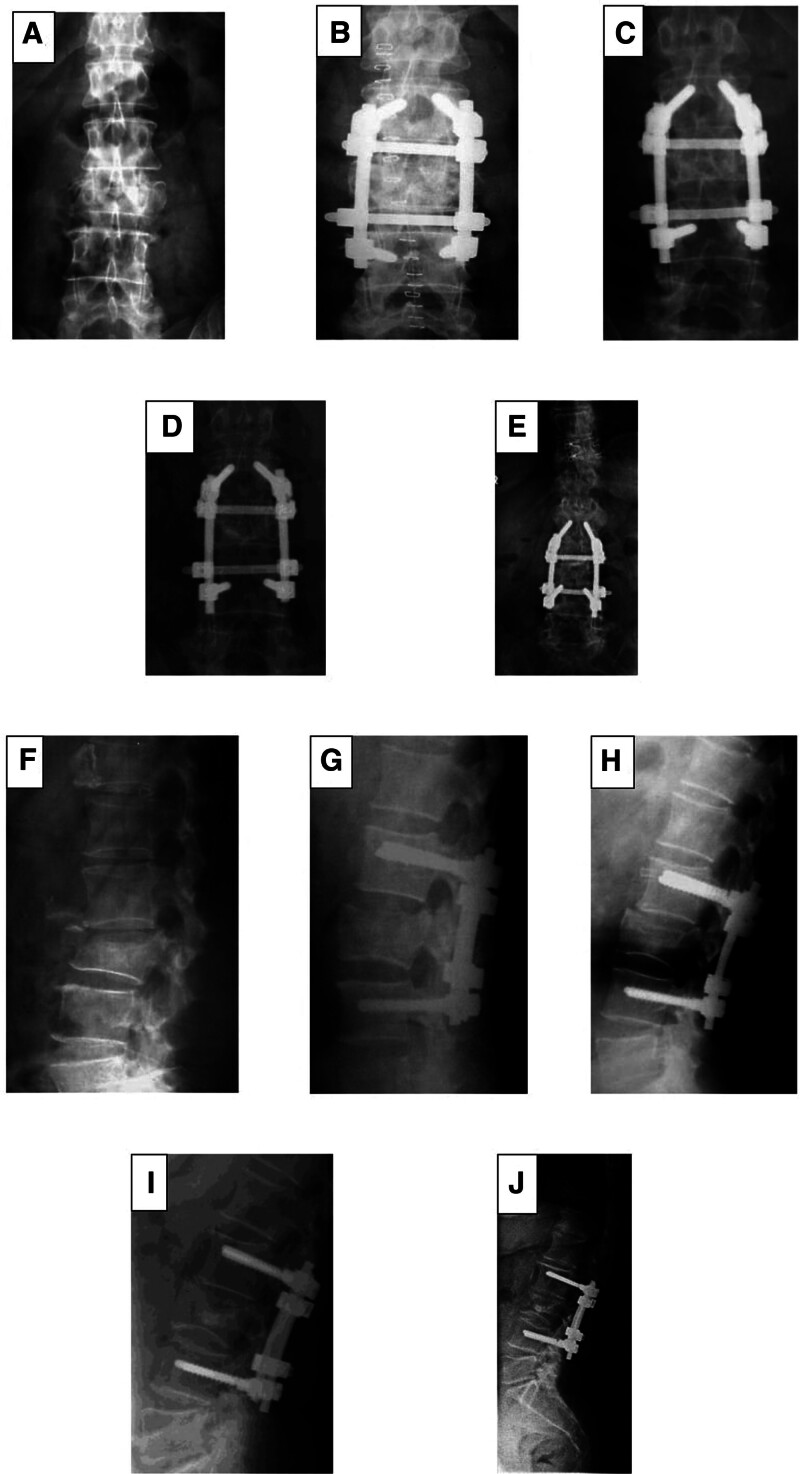
Patient X-ray samples: preoperative AP (A), early postoperative AP (B), 2004 AP (C), 2010 AP (D), 2021 AP (E), preoperative lateral (F), early postoperative lateral (G), 2004 lateral (H), 2010 lateral (I), and 2021 lateral (J). AP = anteroposterior.

**Figure 2.**
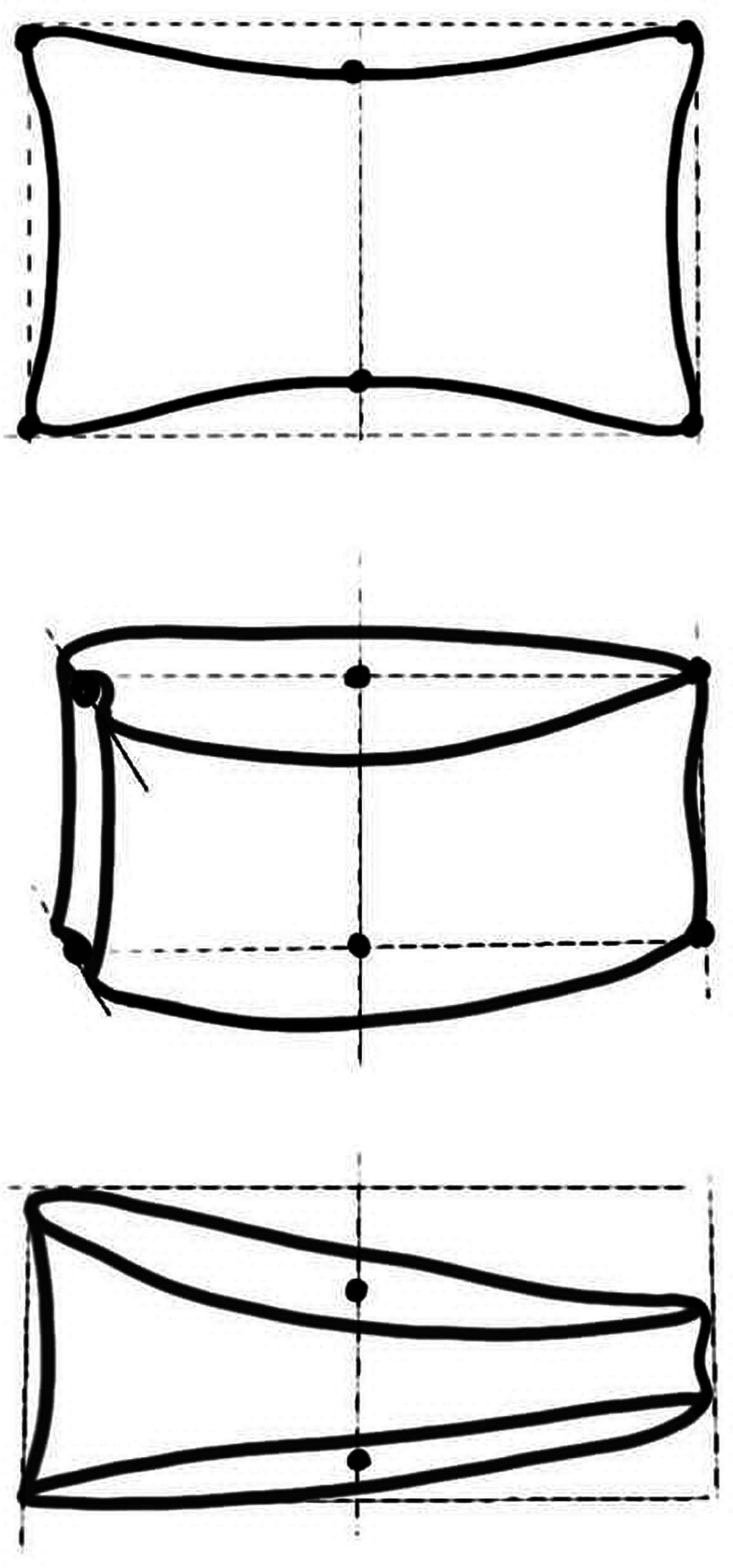
Morphometric measurement of the vertebral body. Six points were identified on radiographs: 4 vertebral corners and 2 endplate midpoints. From Ref.^[24]^

Radiographs taken before surgery, immediately after surgery, and during follow-up were carefully assessed (in 2004, 2010, and at the last follow-up) (Fig. 1). Fractures were classified according to Denis classification.^[4]^ Vertebral body height measurements (anterior, middle, and posterior), including anterior, middle, and posterior and wedge angles, were measured in consensus between 2 different radiologists. Six points were identified on the radiographs: 4 vertebral corners and 2 endplate midpoints (Fig. 2). In cases where the radiograph was not a perfectly lateral view, the selected points represented the midplane of the vertebral body. The midpoints were carefully positioned along a line that equally divided the distal and proximal projections of the superior and inferior endplates, respectively.^[24]^

**Figure 3.**
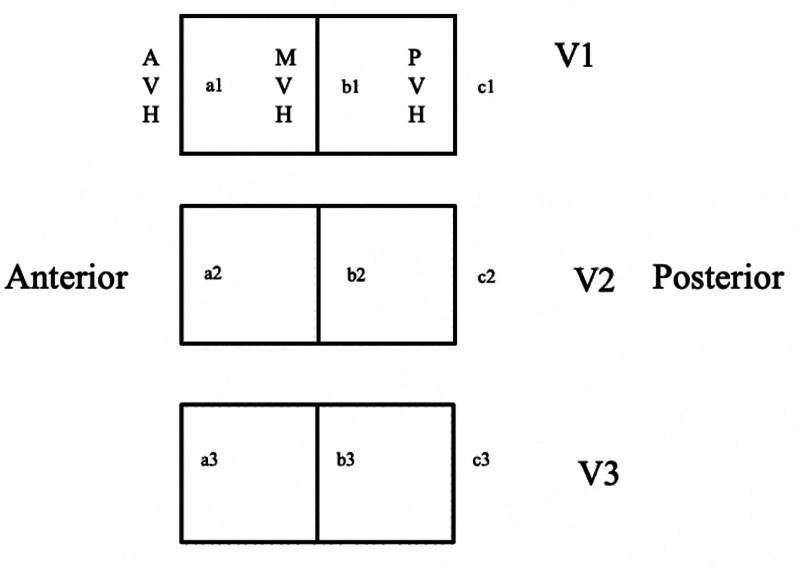
Anterior/middle column vertebral body compression ratio (VBCR) and anterior vertebra body compression percentage (AVBC %) measurement method. From Ref.^[25]^ AVH = anterior column vertebra body height. MVH = middle column vertebral body height, PVH = posterior column vertebral body height, V1 = cranial vertebrae, V2 = fractured vertebrae, V3 = caudal vertebrae, VBCR = a/c, AVBC% = [a2/ (a1 + a3)] × 100.

Loss of vertebral body height was quantified using the anterior/middle column vertebral body compression ratio (VBCR), calculated as the anterior column vertebra body height (AVH) divided by the posterior column vertebra body height (PVH) (VBCR = AVH/PVH). We also measured the percentage of anterior vertebral body compression [AVBC%] and the amount of compression of the anterior vertebral body relative to the average height of the vertebral bodies proximal and distal to the level of injury (Fig. 3). The results of both techniques (VBCR and AVBC%) at each follow-up were compared to detect any loss in vertebral body height.^[25]^ Radiological assessment was performed by 2 senior radiologists, with decisions in case of discrepancy made by consensus.

### 2.4. Statistical analyses

Since the distribution of data was not normal, median and interquartile range values, in addition to mean ± standard deviations, were calculated. Comparisons between more than 2 groups were performed using one-way analysis of variance (ANOVA). A post hoc Dunnett test for repeated measures was performed to compare experimental and control groups. Multiple group comparisons were performed using post hoc Tukey test. Statistical significance was set at *P* < .05. The data are expressed as mean ± SEM. Data were tabulated using Microsoft Excel (Microsoft Corporation, Seattle, WA).

## 3. Results

**Figure 4.**
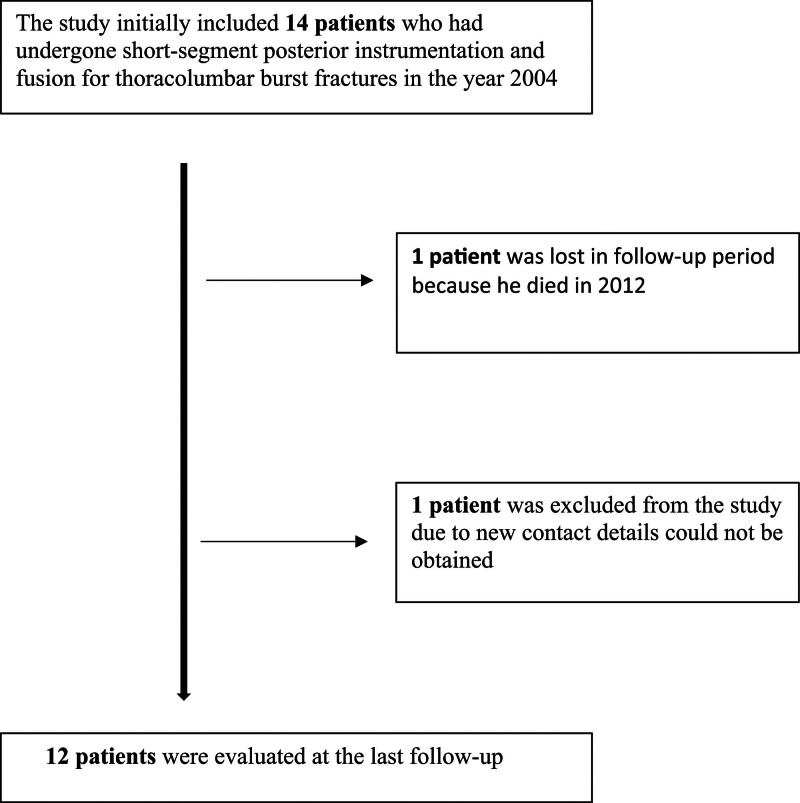
Patient flow chart and exclusion reasons.

Following chart reviews prior to the first study visit in 2004, 14 patients were enrolled in the study. At the last follow-up, 2 patients were unreachable; thus, 12 patients remained in the study: 1 patient had died, and another was excluded because there was no possibility of reaching him since new contact details could not be obtained (Fig. 4). There were 12 patients in this study: 50% male and 50% female patients. The mean age of the patients was 39.7 years of age ranging from 21 to 60 years with a median of 36 years. The mean follow-up period was 225.6 months, ranging from 205 to 265 months, with a median duration of 218 months. Neurological examination results were normal at discharge, and no late complications were reported.

The mean time from trauma to surgery was 8.668 ± 5.1 days (3–16 days) and the median duration was 6 days. The mean hospital stay was 25.1 ± 18.9 days (8–83 days), and the median duration was 21 days. Seven patients were classified as P1 and 5 patients were classified as P2 according to the Denis Pain Scale. According to the Denis Work Scale, 1 patient was classified as W1, 9 as W2, and 2 as W3. The VBCR values did not present statistically significant differences when one-way ANOVA was performed with regard to the follow-up period. Post hoc comparisons using Tukey test did not show any significant differences among the follow-ups either (F (3, 52) = 0.07510, *P* = .973) (Table 1).

**Table 1:** One-way ANOVA for comparison of VBCR.

	SS	DF	MS	F (DFn, DFd)	*P* value
Between follow-ups	46.45	3	15.48	F (3, 52) = 0.07510	*P* = .9731
Within follow-ups	10,721	52	206.2		
Total	10,767	55			

DF = degrees of freedom, DFd = degrees of freedom for the denominator, DFn = degrees of freedom for the numerator, F = F test, MS = mean squares, SS = sum of squares, VBCR = vertebral body compression ratio.

One-way ANOVA indicated no statistically significant differences in AVBC% values across the follow-up periods. Post hoc comparisons using Tukey test did not detect any significant differences between follow-ups (F (3, 52) = 0.1375, *P* = .937) (Table 2).

**Table 2:** One-way ANOVA for comparison of AVBC%.

	SS	DF	MS	F (DFn, DFd)	*P* value
Between follow-ups	67.30	3	22.43	F (3, 52) = 0.1375	*P* = .9372
Within follow-ups	8484	52	163.1		
Total	8551	55			

DF = degrees of freedom, DFd = degrees of freedom for the denominator, DFn = degrees of freedom for the numerator, F = F test, MS = mean squares, SS = sum of squares.

**Figure 5.**
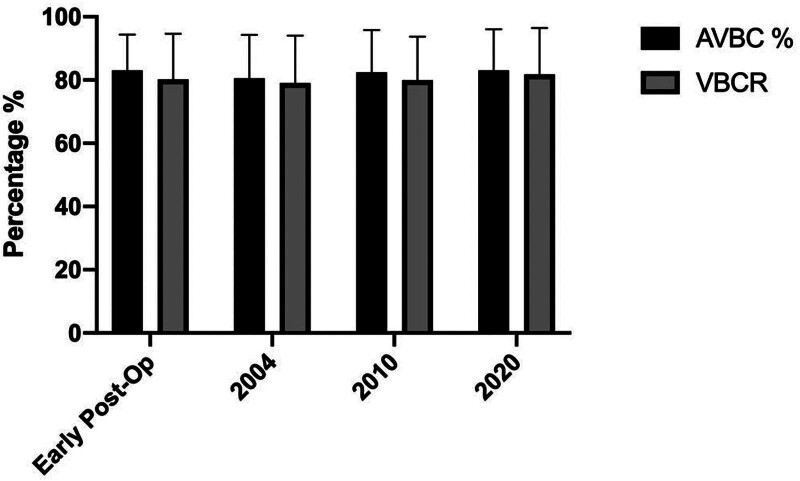
Evaluation of AVBC (anterior vertebra body compression) % and VBCR (vertebral body compression ratio) between follow-up.

There were no statistically significant differences observed in the AVBC% and VBCR measurements across the follow-up periods (*P* = .567, .783, .629, .813) (Fig. 5). However, there was a statistically significant difference between the preoperative and early postoperative wedge angles (*P* < .001). One-way ANOVA did not indicate a statistically significant difference when the wedge angle values between the postoperative follow-ups were compared. No follow-up was significant in post hoc comparisons using Tukey test, F (2.454, 31.91) = 0.1752, *P* = .879 (Table 3). Construct failure was not detected during the last radiological visit.

**Table 3:** One-way ANOVA for comparison of wedge angles.

	SS	DF	MS	F (DFn, DFd)	*P* value
Between follow-ups	0.4821	3	0.1607	F (2.454, 31.91) = 0.1752	*P* = .8793
Within follow-ups	35.77	39	0.9171		
Total	2206	55			

DF = degrees of freedom, DFd = degrees of freedom for the denominator, DFn = degrees of freedom for the numerator, F = F test, MS = mean squares, SS = sum of squares.

## 4. Discussions

In fact, previous studies have demonstrated that the anterior approach, combined anterior and posterior approaches, LSPIF, SSPIF, and even short-segment posterior instrumentation without fusion all showed good efficacy in treating thoracolumbar burst fractures.^[22,26]^ However, no conclusion has been reached regarding the best surgical method for thoracolumbar burst fractures.^[13,27–30]^

Our results showed that wedge angles improved following SSPIF, and this improvement was preserved between short- and long-term follow-ups. It has also been shown that vertebral body height is maintained over a long period after SSPIF.

SSPIF is a common treatment method. This method reduces surgical time and hospital costs and incorporates fewer motion segments in the fusion.^[2,13,29,31–33]^ However, some researchers report drawbacks of SSPIF, which involves a high rate of correction loss over a long period of time, as well as implant failures including screw pullout and breakage.^[32,34–36]^ In contrast, our study demonstrates that SSPIF significantly corrects the loss of anterior and middle corpus height, as well as the inclined wedge angle following a fracture, with this correction being sustained over a 20-year long-term follow-up period.

Indirect decompression and SSPIF accomplish all goals of surgical treatment for thoracolumbar spine fractures.^[36,37]^ Using routine SSPIF, Cho et al^[38]^ treated 70 thoracolumbar burst fractures and described 6° of postoperative kyphosis correction. Carl et al^[14]^ treated 38 thoracolumbar spine fractures using posterior pedicle screw instrumentation and reported an 7° of initial kyphosis correction. Similarly, our findings indicated a significant correction in both the anterior column height (75.8%) and the sagittal wedge angle (7.7°) of fractured vertebrae across all patients immediately following surgical intervention. This underlines the effectiveness of SSPIF in treating deformities resulting from thoracolumbar fractures.

It is commonly thought that SSPIF is a reliable way to achieve good deformity correction in the treatment of thoracolumbar burst fractures. Despite its wide diffusion, SSPIF has been related to a remarkable percentage rate of correction loss, including screw pullout, breakage, and re-kyphosis. Many experts argue that LSPIF offers a reliable alternative for managing thoracolumbar burst fractures, potentially reducing these risks.^[5,34,35]^ Altay et al^[13]^ retrospectively compared the results of SSPIF with those of LSPIF, including 32 thoracolumbar burst fractures treated with SSPIF and 31 treated with LSPIF. Correction loss over 10° occurred in 6 of the 32 patients treated with SSPIF, while this amount of correction loss was found in only 2 out of 31 patients treated with LSPIF. The results indicate lower effectiveness when SSPIF is compared to LSPIF.

Tezeren et al^[39]^ compared the results between short-segment and long-segment instrumentation in 18 thoracolumbar burst fractures and reported that long-segment instrumentation is better than SSPIF in terms of local kyphosis correction and anterior body compression. In contrast, Aly^[40]^ performed a meta-analysis evaluating the efficacy of various levels of fixation with pedicle screw fixation in thoracolumbar burst. The findings indicated no significant difference in kyphosis correction or implant failure rates between short- and long-segment fixations at the final follow-up. However, both techniques showed a progression of kyphosis over time. In our study, sufficient clinical and radiological results were achieved compared with studies in which LSPIF was used. The reduced height of the fractured vertebral body was maintained after a long-term follow-up of 20 years, no kyphotic deformity occurred, and no implant failure was observed.

Many authors have reported a high rate of implant failure in the thoracolumbar region with SSPIF.^[40–42]^ Mc Lain et al^[41]^ treated 19 patients with SSPIF and also suggested that SSPIF was an appropriate method in only the lower lumbar region because of a high rate of failure. Nevertheless, Gelb^[43]^ treated 46 thoracolumbar fractures with SSPIF and found that SSPIF can treat injuries at the thoracolumbar junction, as well as injuries at lower lumbar levels.

Although there were few patients in our series, no statistical differences in the clinical and radiological results between T12-L1 level fractures and other levels of fractures were observed. The improvement in the wedge angle was less in patients whose fracture level was either T12 or L1 compared to those whose fracture levels were either L2, L3, or L4.

We believe that this difference may be related to the bending of the rod to ensure lordosis at the lower levels. However, better corrections at lower levels can be considered as support for the results of McLain et al.^[41]^

The preferential aim of the surgical treatment of thoracolumbar fractures is to achieve pre-trauma functional levels, and radiological correction and physical evaluation are commonly used as outcome criteria.^[40]^ Sanderson et al^[44]^ reported a series of 28 consecutive patients treated for thoracolumbar burst fractures using short-segment pedicle screw fixation. The clinical outcome, based on the Low Back Outcome Score, was rated as excellent in only 50% of cases after an average follow-up period of 3.1 years.

Liao et al^[45]^ performed SSPIF for thoracolumbar fracture treatment and used the Denis pain and work scale for clinical assessment. They found that 60% of patients were P1 and 55% were W1. We similarly used the scales to evaluate the functional status of patients during the follow-up periods; both scales were significantly improved as of the final follow-up (P1 + P2: 100%, W1 + W2: 83.3 %).

All our patients presented with endplate fractures, suggesting that disc degeneration might have been unavoidable. Rigid dorsal fixation and fusion can contribute to stress shielding of the intervertebral disc, potentially leading to biochemical alterations.^[46]^ Moreover, trauma-induced disruption of endplate vascularity has been identified as a significant factor in the development of degenerative disc diseases.^[47,48]^ However, various magnetic resonance imaging (MRI) studies have indicated that while the signal intensity of the discs generally remains unchanged, the most notable alterations are often morphological abnormalities of the disc.^[48]^ Thus, some authors believe that disc space pathology is a result of compression fracture of the osseous endplate.^[2]^ Although it was known that MRI is the best modality for detecting discs, we had signs of any degeneration on our radiographic follow-up.

There are several limitations in the present study: it is a small case series, performed in a retrospective manner, without prospective study design that could analyze the radiological and clinical data available. Therefore, it lacks proper assessment of certain clinical outcomes, as well as a control group where the efficacy of the treatment can be compared with other methods. Moreover, we quantified the bony structures using only plain spinal radiography, and disc pathologies were not evaluated. MRI studies may provide better understanding in both the time course of disc morphological changes and the degree of degenerative disc disease.

## 5. Conclusion

Results of this study showed that SSPIF has the capability to adequately restore anterior height loss, middle corpus height loss, and wedge angle inclination after vertebral fracture. Moreover, these corrections were sustained over the 20-year follow-up period. Therefore, it can be considered an effective and safe mode of treatment for thoracolumbar burst fractures, and is among the best options in comparison with other modes of treatment.

## Acknowledgments

We would like to thank our secretary, Mr. Omer Ulku Ilkay, because he helped us so much while accessing the old archives that posed some problems. We confirm that Mr. Omer Ulku Ilkay has given permission to be named in this section.

## Author contributions

**Conceptualization:** Yigit Kultur, İlker Sarikaya, Onder Aydingoz.

**Data curation:** Yigit Kultur, İlker Sarikaya, Mahmut Kursat Ozsahin, Onder Aydingoz.

**Formal analysis:** Yigit Kultur, Cumhur Deniz Davulcu, Onder Aydingoz.

**Investigation:** Yigit Kultur, İlker Sarikaya, Onder Aydingoz.

**Methodology:** Yigit Kultur, İlker Sarikaya, Onder Aydingoz.

**Project administration:** Yigit Kultur, İlker Sarikaya, Mahmut Kursat Ozsahin, Cumhur Deniz Davulcu, Onder Aydingoz.

**Resources:** Yigit Kultur, İlker Sarikaya, Onder Aydingoz.

**Software:** Yigit Kultur, İlker Sarikaya, Mahmut Kursat Ozsahin, Cumhur Deniz Davulcu, Onder Aydingoz.

**Supervision:** Cumhur Deniz Davulcu, Onder Aydingoz.

**Validation:** Yigit Kultur, Cumhur Deniz Davulcu, Onder Aydingoz.

**Visualization:** Cumhur Deniz Davulcu, Onder Aydingoz.

**Writing – original draft:** Yigit Kultur, İlker Sarikaya, Mahmut Kursat Ozsahin, Onder Aydingoz.

**Writing – review & editing:** Yigit Kultur, İlker Sarikaya, Mahmut Kursat Ozsahin, Onder Aydingoz.
